# Synthesis of High-Performance and Biodegradable Polymer Blends Based on Poly(butylene succinate) and Grafted Polyrotaxane via Controlled Reactive Processing

**DOI:** 10.3390/polym18010038

**Published:** 2025-12-23

**Authors:** Yuki Kitada, Akira Ishigami, Yutaka Kobayashi, Yoshiyuki Suetsugu, Hironori Taguchi, Takako Kikuchi, Hiroshi Ito

**Affiliations:** 1 Graduate School of Organic Materials Science, Yamagata University, 4-3-16 Jonan, Yonezawa 992-8510, Japan; t241055m@st.yamagata-u.ac.jp; 2 Research Center for GREEN Materials and Advanced Processing (GMAP), Yamagata University, 4-3-16 Jonan, Yonezawa 992-8510, Japan; kobayashi.y@yz.yamagata-u.ac.jp (Y.K.); suetsugu@yz.yamagata-u.ac.jp (Y.S.); 3 Chemicals Evaluation and Research Institute, Japan (CERI), 1600 Shimotakano, Sugimoto-machi, Saitama 345-0043, Japan; taguchi-hironori@ceri.jp (H.T.); kikuchi-takako@ceri.jp (T.K.)

**Keywords:** transesterification, urethanization, grafted polyrotaxane, poly(butylene succinate), hexamethylene diisocyanate, polymer blends, impact strength, toughness

## Abstract

In this study, novel, high-strength polymer blends were synthesized using poly(butylene succinate) (PBS) modified with grafted polyrotaxane (GPR). Then, their mechanical properties and morphologies were evaluated. A unique, two-step, reactive kneading method was developed to substantially improve the mechanical properties of PBS, which promoted transesterification reaction using an organo-titanium catalyst (Ti) in the first step and a urethanization reaction using hexamethylene diisocyanate (HDI) in the second step. The optimized blend material, [PBS/GPR10/Ti]-HDI, achieved remarkable toughening, and its Izod impact strength increased approximately seven-fold compared with that of unmodified PBS. Scanning electron microscopy (SEM) of the fracture surfaces confirmed a transition from brittle to ductile fracture, attributed to the controlled reaction sequence. First, strong chemical bonds formed at the PBS/GPR interface via Ti-catalyzed transesterification. Then, HDI induced simultaneous internal crosslinking (gelation) of the GPR domains and chain extension of the PBS matrix. This modification strategy maintained the excellent inherent soil biodegradability of PBS while improving its degradability in marine environments. This study presents a new guideline for designing materials that can considerably enhance the mechanical properties of biodegradable plastics.

## 1. Introduction

Plastic products are widely used in our daily lives because they are lighter, corrosion-resistant, durable, and cost-effective compared with metals. Thus, plastics have replaced metals, glass, and wood across various areas such as consumer goods and automotive, medical, and aerospace industries [1,2,3,4,5,6]. According to an OECD report [7], plastic usage increased nine-fold in regions across ASEAN countries, Japan, China, and South Korea between 1990 and 2022, reaching 152 million tons in 2022 and accounting for approximately one-third of the total global usage. The global plastics industry generated ~1.6 trillion USD in 2019, equivalent to ~2% of the world’s GDP; the plastics market is projected to continue expanding [8,9,10]. However, plastic waste accumulation in the environment, particularly marine plastic pollution, has emerged as a serious issue. A 2021 UNEP report reported that ~9–14 million tons of plastic waste entered aquatic environments annually by 2016, which accumulated in marine ecosystems without degrading. Fish and seabirds ingested these plastic fragments, causing their death and disrupting ecological balance. Microplastics have entered the food chain, impacting human health [11,12,13,14,15,16]. Waste disposal and environmental pollution have caused considerable economic losses, necessitating the urgent development of sustainable materials [17].

As such, biodegradable plastics have garnered attention as sustainable materials. Microorganisms and enzymes degrade these plastics into water and carbon dioxide, resulting in a shorter residence time in the environment compared with conventional petroleum-derived plastics. Therefore, these materials contribute to the development of a sustainable society [18,19]. Representative biodegradable plastics include polylactic acid (PLA), polyhydroxyalkanoates (PHA), and poly(butylene succinate) (PBS) [20,21,22,23,24], with unique properties. In particular, PBS is a highly moldable, heat-resistant material with wide practical use. However, its applicability is limited by its comparable mechanical properties to those of general-purpose resins. Despite their advantages, PLA is brittle and PHA has low heat resistance and cannot independently meet the required performance standards. Therefore, plastics with enhanced mechanical properties and biodegradability have been fabricated using methods such as blending, copolymerization, and filler addition [25,26,27,28,29,30]. However, the enhanced properties are accompanied by deteriorated biodegradability or processability [31].

Among all the aforementioned methods, polymer blending effectively improves the mechanical properties of biodegradable polymers [32]. By combining materials such as PBS with flexible and tough polymers, improvements in mechanical properties can be achieved contrary to that with a single material. For example, Jamaluddin et al. reported that blending PBS with thermoplastic polyurethane improved toughness and elongation at break while maintaining tensile strength [30]. Additionally, Hu et al. demonstrated that blending PBS with a bio-based elastomeric copolymer (PBBS) effectively enhanced toughness, including impact resistance, through tunable compatibility [33]. Beyond these conventional strategies, supramolecular chemistry has attracted attention as a novel polymer design methodology in recent years.

Polyrotaxane (PR) is a supramolecular polymer containing multiple cyclic molecules such as cyclodextrins threaded onto a linear axial polymer, wherein both ends of the axis are capped by bulky molecules [34,35]. This structure exhibits a “pulley effect”, whereby the cyclic molecules can move freely along the axis. Thus, PR can disperse and absorb external stress at the molecular level, imparting high toughness to the material [36,37,38,39]. Grafted polyrotaxane (GPR) contains polymer chains (polycaprolactone or PCL) that are grafted onto the cyclic molecules of PR [40,41]. GPR can enhance the toughness of brittle polymers such as PBS by modifying their structure. However, physical blending leads to weak interfacial interactions between the two polymers, resulting in phase separation and stress concentration at the interface [32] that limit the improvement in mechanical properties. The pulley effect of GPR can be further enhanced via optimal molecular-level arrangement and strong bonding with the PBS matrix. In addition, intermolecular reactions can be chemically controlled via reactive kneading technology for enhancing the mechanical properties.

Herein, this technology was used to synthesize a PBS/GPR blend that considerably enhances the toughness of PBS, thereby enhancing its applicability to challenging, high-value fields such as durable industrial components and reusable consumer goods. The mechanism underlying enhancements in the mechanical properties were elucidated via property evaluations and higher-order structural observations. Additionally, the impact of GPR blends on biodegradability was assessed. This study aims to establish new guidelines for designing materials that considerably enhance the mechanical properties of PBS while maintaining its excellent biodegradability.

## 2. Experimental

### 2.1. Materials

**Figure 1:**
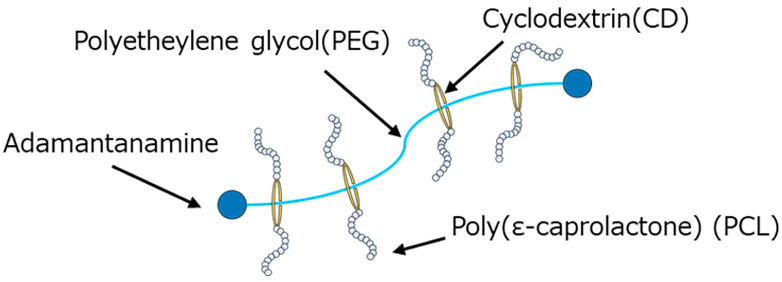
Chemical structure of GPR.

PBS (FZ91PB, Mitsubishi Chemical Corporation) was used as the matrix resin for polymer blend materials. GPR with PCL side chains (SH3400P, Advanced Soft Materials, Inc., Ibaraki, Japan) was used to modify the mechanical properties of PBS. Figure 1 shows the chemical structure of GPR. To improve the reactivity and miscibility of GPR with PBS, an organo-titanium compound (TBT TA-21, Matsumoto Fine Chemicals Co., Ltd., Chiba, Japan) was used as the transesterification catalyst, and hexamethylene diisocyanate (HDI, Tokyo Chemical Industry Co., Ltd., Tokyo, Japan) was used as the crosslinking agent. Before use, PBS was dried in a vacuum oven at 80 °C for 4 h and GPR was dried for 8 h.

### 2.2. Sample Preparation

Table 1 shows the composition ratios of the first-step blends of PBS, GPR, and additives. Further, Table 2 shows the composition ratios of the second-step blends with sequentially added additives based on first-step blends. Melt kneading was performed in a twin-screw melt-kneading extruder (KZW15TW-45MG-NH (-700), Technovel Corporation, Osaka, Japan) with an L/D ratio of 45 and a screw diameter of 15 mm. To investigate the effect of reaction sequence on structure formation, samples were prepared by changing the order of introducing additives (Table 2). For instance, [PBS/GPR5/Ti]-HDI indicates a blend of 95 wt% PBS, 5 wt% GPR, and 0.1 phr Ti catalyst was kneaded in the first step, followed by the addition of 1 phr HDI in the second step.

Based on preliminary experiments conducted with GPR contents up to 20 wt%, the impact strength was observed to saturate, exhibiting values comparable to those at 10 wt%. Therefore, the maximum GPR content was set at 10 wt% in this study.

**Figure 2:**
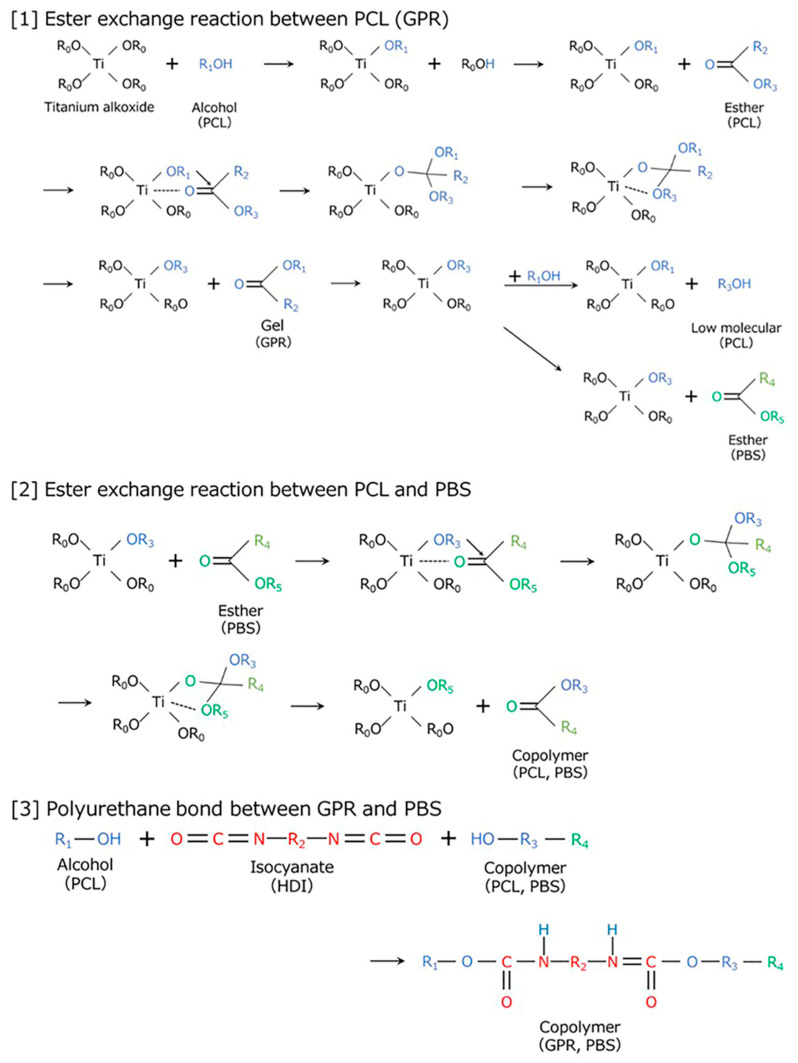
Two-step kneading process for [PBS/GPR5/Ti]-HDI and [PBS/GPR10/Ti]-HDI.

Figure 2 shows the reaction scheme for the two-step kneading method used for synthesizing [PBS/GPR5/Ti]-HDI and [PBS/GPR10/Ti]-HDI. PBS and GPR were first kneaded in the presence of a Ti catalyst to graft the PCL side chains of GPR onto PBS via transesterification. HDI was added in the second kneading step, which reacted with the terminal hydroxyl groups of PCL and PBS, thereby crosslinking GPR and chemically bonding it to PBS. As the PCL side chains of GPR contain higher number of reactive sites and are more flexible than the linear PBS main chain, first-step transesterification and second-step urethanization likely proceeded preferentially on the PCL side chains [42,43,44].

**Figure 3:**
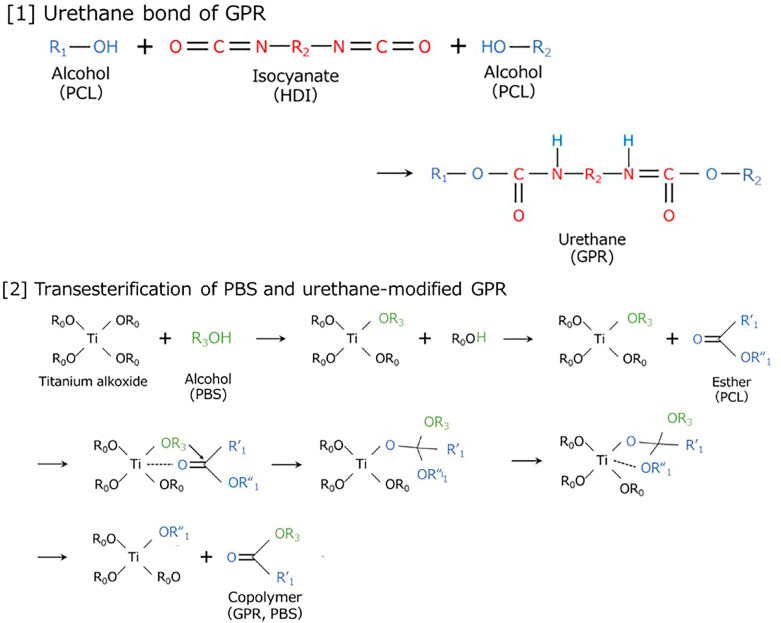
Two-step kneading process for [PBS/GPR10/HDI]-Ti.

Figure 3 shows the reaction scheme for the two-step kneading method used to synthesize [PBS/GPR10/HDI]-Ti. The PCL side chains of GPR first underwent self-crosslinking and gelation via urethane bond formation with HDI. A Ti catalyst was introduced in the second step, which promoted transesterification between the remaining reactive sites and PBS after the concentration of PCL hydroxyl groups decreased.

**Figure 4:**
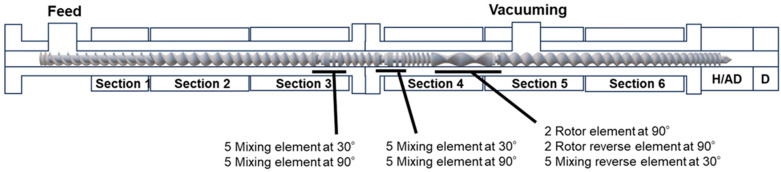
Screw configuration and positions of Sections 1–6, H/AD, and D.

Figure 4 shows the screw design and temperature settings for each section of the extruder. The one-step kneaded samples were prepared at a feed rate of 0.6 kg/h and a screw speed of 100 rpm (Table 1), whereas the two-step kneaded samples were prepared at a feed rate of 0.4 kg/h and a screw speed of 100 rpm (Table 2). These processing parameters were optimized to allow for sufficient urethanization reaction time while preventing excessive crosslinking, which could compromise processability. A vent was installed in Section 4 and connected to a vacuum pump to expel gases generated during the reaction. A 60-mesh screen was placed between the extruder head (H/AD) and the die (D) to maintain pressure within the molten resin. The temperature in Section 4 increased beyond the set value due to shear heating caused by the high melt viscosity of PBS. Table 3 shows the set temperatures for each sample preparation, and Table 4 shows the extruder head pressure and the actual temperature measured in Section 4. Regarding the reaction temperature, Matsumoto et al. reported that TBT effectively catalyzes ester-exchange reactions at temperatures ranging from 120 °C to 150 °C [45]. In this study, although the barrel temperature was set to 135 °C, the actual resin temperature reached 135–143 °C due to shear heating (Table 4). This indicates that the processing temperature was sufficient for TBT to function as a transesterification catalyst.

The resulting pellets were molded into rectangular specimens of 64 × 12.7 × 3.2 mm^3^ for impact testing using a vertical injection molding machine (Mold Lock X-801, Century Innovation Corporation, Tokyo, Japan) under these conditions: an injection temperature of 190 °C, a mold temperature of 40 °C, and an injection speed of 1.36 cm^3^/s. For tensile testing, ~100-µm-thick films were prepared using a hot-press machine (IMC-11FD, Imoto Machinery Co., Ltd., Kyoto, Japan) at 160 °C for 5 min under 15 MPa in vacuum. The films were then cooled rapidly under atmospheric conditions in a second hot-press machine (MP-WC, Toyo Seiki Seisaku-Sho, Ltd., Tokyo, Japan) at 20 °C and 15 MPa of pressure.

### 2.3. Characterization

#### 2.3.1. Fourier Transform-Infrared Spectroscopy

Fourier transform-infrared spectroscopy (FT-IR) measurements were performed using an IRAffinity-1S Fourier transform-infrared spectrophotometer (Shimadzu Corporation, Kyoto, Japan). Samples were characterized using the attenuated total reflectance method in the wavenumber range of 2000–400 cm^−1^, with a resolution of 1 cm^−1^ and 40 scans.

#### 2.3.2. Differential Scanning Calorimetry

The thermal properties of the PBS blend system, one-step kneaded samples, and two-step kneaded samples were analyzed via differential scanning calorimetry (DSC, DSC 250, TA Instruments Japan Inc., Tokyo, Japan) Measurements were performed on pellet samples (approximately 5–10 mg) over a temperature range of −50 °C to 150 °C at a heating rate of 10 °C/min. All DSC measurements were performed under a nitrogen atmosphere. To evaluate the inherent thermal properties of the chemically modified materials by eliminating the thermal history formed during processing, data from the second heating scan were used for analysis.

#### 2.3.3. Rheological Properties

The rheological behavior of one-step kneaded samples and two-step kneaded samples was measured using a rotational rheometer (MCR 302, Anton Paar Japan K.K., Tokyo, Japan). Dynamic viscoelasticity was measured using parallel plates (25-mm diameter) with a 1-mm gap at a temperature of 135 °C and an angular frequency range of 0.1–100 rad/s. Measurements were conducted within the linear viscoelastic region (10% strain) under a nitrogen atmosphere.

#### 2.3.4. Thermogravimetric Analysis

The thermal decomposition properties of the PBS blends, one-step kneaded samples, and two-step kneaded samples were evaluated using thermogravimetric analysis (TGA, Q50, TA Instruments Japan Inc., Tokyo, Japan). Measurements were conducted from 22 °C to 500 °C at a heating rate of 10 °C/min under a nitrogen atmosphere. The decomposition temperature (T_d,5%_) was defined as the temperature at which the weight loss reached 5%. The maximum decomposition temperature (T_max_) was determined from the peak temperature of the derivative thermogravimetry (DTG) curve.

#### 2.3.5. Dynamic Mechanical Analysis

Solid-state viscoelasticity was measured via dynamic mechanical analysis (DMA) (RSA G2, TA Instruments, Inc.) in the tensile mode on rectangular specimens (~6.5-mm wide and 20-mm long). These measurements were performed at a frequency of 1 Hz and dynamic and static stresses of 13 N from −80 °C to 80 °C at a heating rate of 5 °C/min.

#### 2.3.6. Transmission Electron Microscopy

The morphology of blend materials was observed via transmission electron microscopy (TEM, JEM-2100F, JEOL Ltd., Tokyo, Japan). The PCL side chains of GPR, which have a lower electron density than PBS, were selectively stained with ruthenium tetroxide (RuO_4_) vapor. Then, ~100-nm-ultrathin sections were prepared using an ultramicrotome (Ultracut-UCT, Leica Microsystems, Tokyo, Japan) for TEM observations at an accelerating voltage of 200 kV.

### 2.4. Mechanical Properties

#### 2.4.1. Impact Strength Test

Notched Izod impact tests were performed in accordance with the ASTM D256 standard using an impact tester (IT, Toyo Seiki Seisaku-Sho, Ltd., Tokyo, Japan) equipped with a 5.5 J hammer. The rectangular specimens were notched 2-mm deep using an automatic notching machine (notching tool A-4E, Toyo Seiki Seisaku-Sho, Ltd., Tokyo, Japan), and the measurements for 10 specimens were averaged to obtain the final values.

#### 2.4.2. Morphological Observation of Fractured Surfaces

After the Izod impact test, the fracture surfaces of specimens were observed using a scanning electron microscope (JSM-IT800 (SHL), JEOL Ltd., Tokyo, Japan). For comparison, specimens not subjected to impact testing were immersed in liquid nitrogen for 5 min and immediately subjected to brittle fracture perpendicular to the notch face. The resulting fracture surfaces were then observed.

#### 2.4.3. Tensile Tests

Tensile tests on hot-pressed films were conducted at a tensile speed of 5 mm/min using a universal testing machine (Strograph VGS1-E, Toyo Seiki Seisaku-Sho, Ltd., Tokyo, Japan). The specimens were die-cut into dumbbell shapes (overall length: 35 mm, gauge length: 10 mm, parallel width: 2 mm, grip width: 6 mm, thickness: ~100 µm). Toughness (W_f_) was calculated by numerically integrating the area under the stress–strain curve. The stress (*σ*(*ε*)) up to the strain at break (*ε_b_*) was integrated using Equation (1):

(1) $$W_{f}=\int_{o}^{\varepsilon_{b}}\sigma \left(\varepsilon \right)d\varepsilon$$

### 2.5. Gel Fraction Measurement

To measure the gel fraction, ~5.0 g of the sample was weighed and immersed in 500 mL of chloroform. It was then left to stand at 80 °C for 1 h to extract the insoluble material. The solvent was removed after extraction, and the residue was washed several times with fresh solvent. The residue was dried in a hot-air oven at 80 °C for 1 h, and its weight was measured. The gel fraction was calculated using Equation (2):

(2) $$Gel\;fraction(\%)=\frac{W_{dry}}{W_{initial}}\times 100$$

where *W_initial_* is the initial weight of the sample and *W_dry_* is the weight of the insoluble material after chloroform immersion and drying.

### 2.6. Biodegradability Test

#### 2.6.1. Biochemical Oxygen Demand Test

For the biochemical oxygen demand (BOD) test, a 250-mL glass-batch-type test apparatus was used (BOD measuring device, OxiTop method test apparatus, Xylem Japan K.K.). This apparatus measured the pressure change inside a sealed container to continuously determine the oxygen consumption associated with the microbial decomposition of organic matter. Carbon dioxide generated during decomposition was absorbed by sodium hydroxide (NaOH) inside the container [46,47,48].

Oxygen consumption (BOD, mg/L) was calculated using Equation (3):

(3) $${BOD}_{t}=\frac{\left(p_{0}-p_{t}\right)\cdot V_{m}}{R\cdot T\cdot V_{s}}$$

where *p*_0_ is the initial pressure (Pa), *p_t_* is the pressure at time *t* (Pa), *V_m_* is the molar volume (m^3^/mol), *R* is the gas constant (Pa·m^3^/mol·K), *T* is the absolute temperature (K), and *V_s_* is the volume of the test solution (L).

The percentage of biodegradation (*%*) was calculated by dividing the difference in oxygen consumption between the sample and blank test (*BOD_blank_*) with the theoretical oxygen demand (*ThOD*) of each sample (Equation (4)):

(4) $$Biodegradation\left(\%\right)=\frac{{BOD}_{t}-{BOD}_{blank}}{ThOD}\times 100$$

The degradation behaviors of the positive control of cellulose and GPR were compared by measuring CO_2_ evolution in a separate test system. The cumulative CO_2_ evolution for cellulose and GPR increased over time and plateaued after their respective number of days. To compare the degradation rates of samples, the final plateau value was considered its maximum degradation (100%); then, the time taken to reach this value was calculated.

Seawater and sediment collected from Sagami Bay in Kanagawa Prefecture were used as the microbial sources for this test. The seawater was filtered through a 420-10 filter bag (As One Corporation, Osaka, Japan), and the sediment was passed through a 2-mm mesh sieve. To prepare microbe-containing extracted seawater, 100 g of sediment and 600 mL of seawater were placed in a 1 L glass bottle; this mixture was sonicated for 10 s, thoroughly stirred, and then filtered again through the filter bag. Before the test, nitrogen (N) and phosphorus (P) sources were added to maintain microbial activity.

#### 2.6.2. Disintegration Test

For the disintegration test, the extracted seawater and film samples were placed in 500-mL glass bottles; their residual weights were measured after 14, 28, 42, and 56 days. To facilitate recovery after degradation, the samples were sandwiched between polyethylene meshes and immersed in 200 mL of extracted seawater. The bottles were placed in a chamber with constant temperature and humidity (KCL-2000W, Tokyo Rikakikai Co., Ltd., Tokyo, Japan) at 20 °C for 56 days. These were then shaken daily to disperse the sediment. Three tests (n = 3) were performed for each sample.

The samples were also evaluated for their degree of disintegration in soil. To this end, the films were buried in pots containing soil collected from the surface layer of a field in Sugito-machi, Kitakatsushika-gun, Saitama Prefecture. The residual weight was measured after 14, 28, 42, and 56 days. Then, the films were removed and washed in a solution of 25 g of sodium dodecyl sulfate (SDS) dissolved in 300 mL of distilled water to remove the formed biofilm. The samples were vacuum-dried in an AVO-310 vacuum dryer (As One Corporation) at 80 °C for more than 12 h and subsequently weighed.

## 3. Results and Discussion

### 3.1. FT-IR Analysis of Chemical Structures

**Figure 5:**
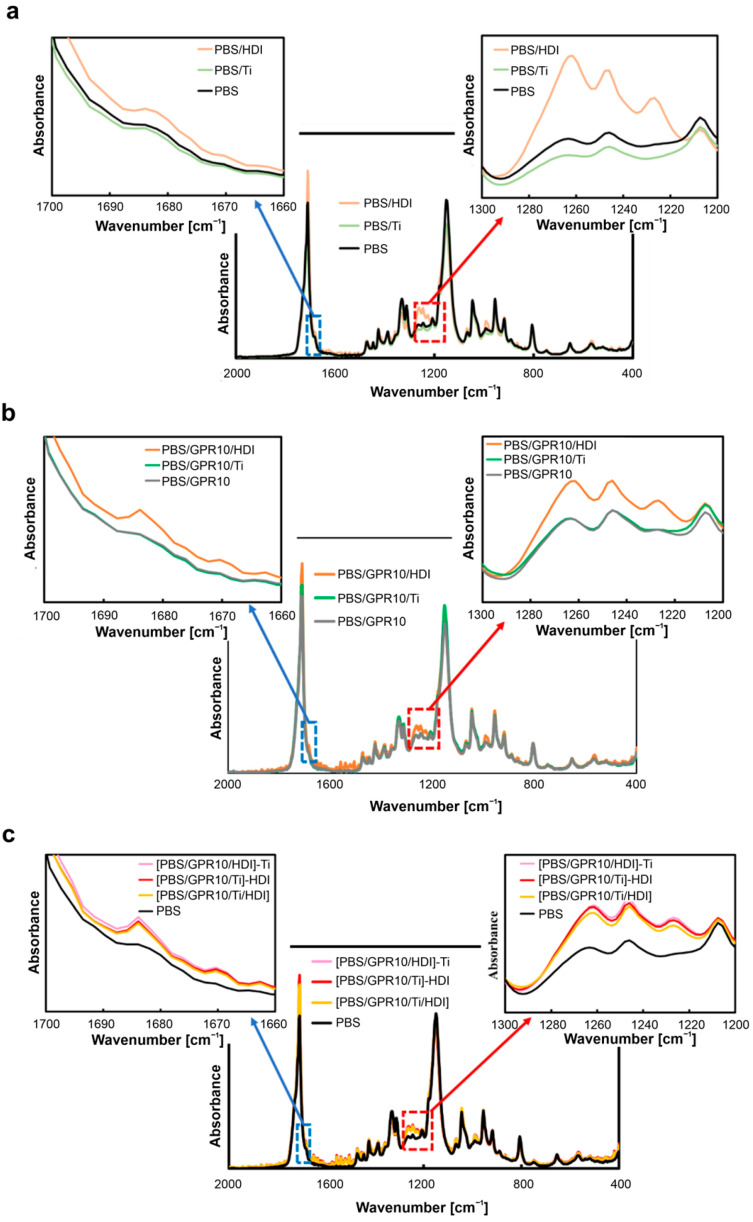
FT-IR spectra showing urethane bond formation: (**a**) PBS, PBS/Ti, and PBS/HDI; (**b**) GPR-containing samples; and (**c**) [PBS/GPR10/Ti/HDI] synthesized via two-step kneading.

Changes in the chemical structures of samples during reactive kneading, particularly the formation of urethane bonds upon adding HDI, were verified via FT-IR measure-ments. Figure 5 shows the corresponding spectra. The pronounced change in spectral features, particularly in the 1200–1270 cm^−1^ region, confirms the formation of urethane bond. The spectrum of PBS shows absorption peaks around 1245 and 1265 cm^−1^, whereas that of PBS/GPR10 shows an additional peak around 1220–1230 cm^−1^, corresponding to GPR.

The peak intensity increased upon adding HDI. For instance, the peak intensities at 1245 and 1265 cm^−1^ are considerably higher for PBS/HDI than those for PBS. The FT-IR spectra of PBS/GPR10/HDI and [PBS/GPR10/Ti]-HDI show peaks at 1220–1230 and 1245 cm^−1^, respectively, with considerably higher intensity than that of PBS/GPR10. This consistent increase in peak intensity indicates that the absorption band derived from coupled C-N stretching and N-H bending vibrations of the newly formed urethane bonds is superimposed on the existing peaks corresponding to PBS and GPR, respectively [49,50,51,52,53].

In the region around 1680 cm^−1^, the shoulder peak intensity considerably increases for HDI-containing samples. This possibly results from the superposition of C=O stretching vibration of the urethane bond. The selective increase in the intensity of specific peaks upon adding HDI strongly indicates that the isocyanate groups of HDI reacted with the terminal hydroxyl groups on the PCL side chains of GPR and PBS during reactive kneading, forming urethane bonds [52,53,54,55].

### 3.2. Thermal Properties Evaluated via DSC

**Figure 6:**
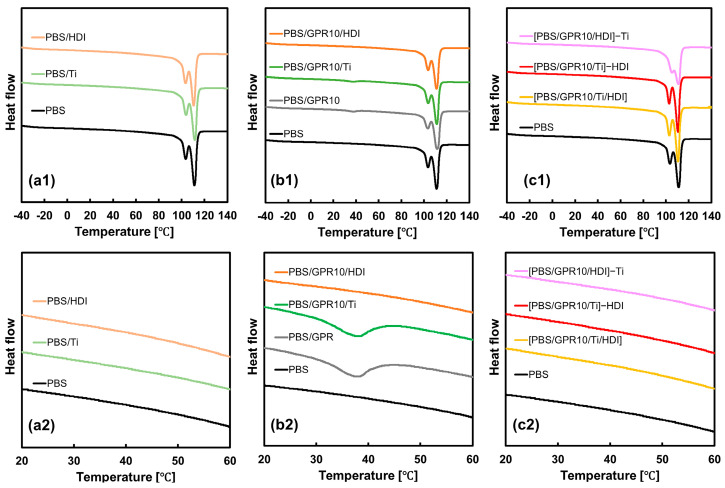
DSC thermograms of (**a**) PBS, PBS/Ti, and PBS/HDI; (**b**) GPR-added samples; and (**c**) [PBS/GPR10/Ti/HDI] synthesized via two-step kneading. The plots labeled (**a1**,**b1**,**c1**) show the full temperature range (−40 °C to 140 °C), whereas those labeled (**a2**,**b2**,**c2**) show a magnified view of the 20–60 °C range.

Figure 6 and Table 5 show the glass-transition temperature (T_g_) and melting point (T_m_) of samples obtained via DSC measurements. The crystallinity of PBS in PBS/Ti, PBS/GPR10, and PBS/GPR10/Ti did not change considerably compared with unmodified PBS, indicating that the crystalline structure of PBS remained largely intact. In contrast, the crystallinity of PBS/HDI considerably decreased, possibly due to chain-extension reaction with HDI. This increased the molecular weight, reducing polymer chain mobility, and hindered PBS crystallization. The T_m_ of PBS/GPR10 and PBS/GPR10/Ti corresponding to the PCL side chains of GPR was ~40 °C, indicating that GPR formed phase-separated domains within the matrix, inside which PCL crystallized. The melting enthalpy of PCL in PBS/GPR10/Ti was lower than that in PBS/GPR10, suggesting that transesterification proceeded due to the Ti catalyst. This reaction caused PCL chains to bond with each other, thereby hindering their crystallization.

In the DSC thermograms of [PBS/GPR10/Ti/HDI] and the two-step kneaded samples ([PBS/GPR5/Ti]-HDI and [PBS/GPR10/Ti]-HDI), the melting peak associated with GPR disappeared. This was possibly because urethanization proceeded efficiently, crosslinking the PCL side chains with the PBS and completely suppressing crystallization. In contrast, the T_g_ of PBS in each sample shifted to a lower temperature compared with that of unmodified PBS. In HDI-containing samples, the introduction of flexible aliphatic methylene chains (–(CH_2_)_6_–) improved molecular chain mobility in the amorphous region and decreased T_g_ [55,56]. The low-molecular-weight components, i.e., the byproducts of the transesterification, in Ti-containing samples may have acted as plasticizers.

### 3.3. Rheological Properties Evaluation

**Figure 7:**
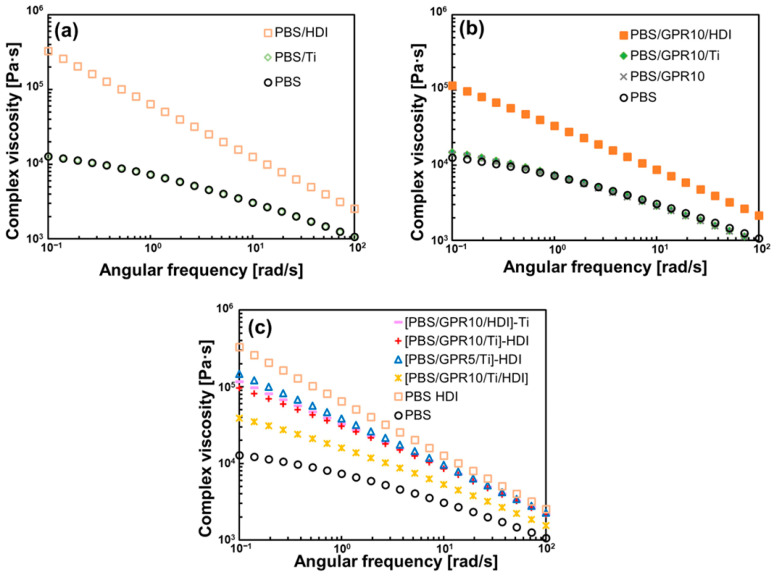
Frequency dependence of complex viscosity for (**a**) PBS, PBS/Ti, and PBS/HDI; (**b**) GPR-added samples; and (**c**) [PBS/GPR10/Ti/HDI] synthesized via two-step kneading.

Figure 7 shows the melt viscoelasticity measurements obtained with a rotational rheometer. The complex viscosity (η*) of PBS/Ti and PBS/GPR10 did not change considerably compared with that of PBS. In contrast, the η* of HDI-containing samples increased considerably, particularly in the low-frequency region. These results confirmed the occurrence of PBS chain extension and GPR crosslinking by HDI, which increased the molecular weight of the sample [57,58].

Interestingly, [PBS/GPR10/Ti]-HDI exhibited a lower viscosity than [PBS/GPR5/Ti]-HDI. The introduction of branched structures generally promotes molecular chain entanglement and increases viscosity under shear flow. However, long-chain branching can hinder molecular chain entanglement, thereby reducing the fraction of chains that contribute to viscosity enhancement [59,60,61]. These findings indicated that two-step kneading formed a branched structure, in which PBS was grafted onto the side chains of GPR that hindered molecular chain entanglement and decreased viscosity.

### 3.4. DMA of Solid-State Viscoelasticity

**Figure 8:**
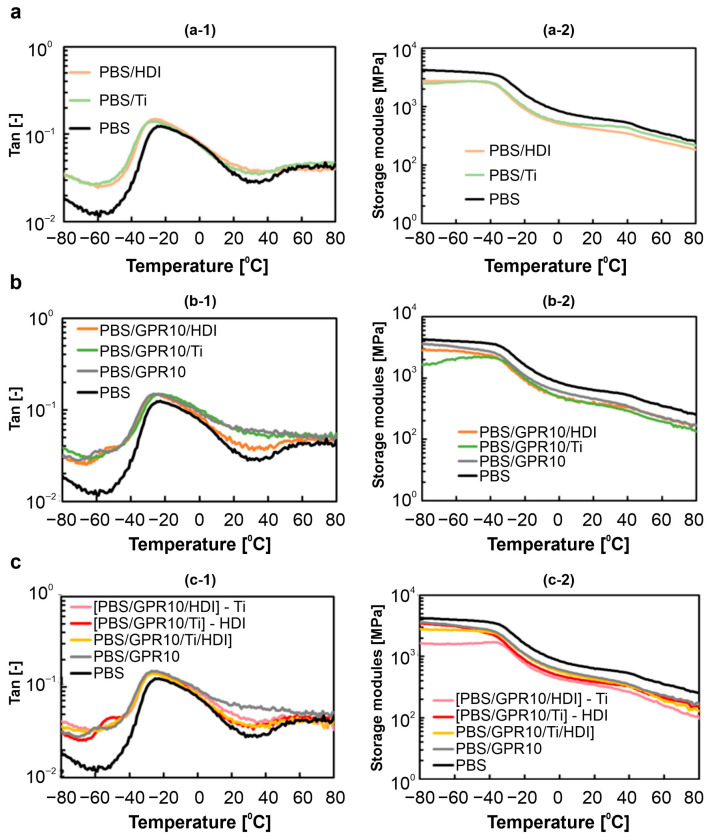
Temperature dependence of (**a-1**,**b-1**,**c-1**) tan δ and (**a-2**,**b-2**,**c-2**) storage modulus (E′) for (**a**) PBS, PBS/Ti, and PBS/HDI; (**b**) GPR-added samples; and (**c**) [PBS/GPR10/Ti/HDI] synthesized via two-step kneading.

Figure 8 shows the tan δ and storage modulus (E′) curves obtained from DMA measurements for each sample. Table 6 shows the corresponding T_g_ determined from the peak positions of tan δ curves.

Unmodified PBS exhibited a single tan δ peak corresponding to its amorphous phase around −30 °C. This peak shifted to lower temperatures in the curves for the PBS/Ti and PBS/HDI samples, exhibiting a similar trend to the melting-point depression observed via DSC. The E′ of the PBS/Ti sample was lower than that of unmodified PBS in the glassy state (T < T_g_). This indicated that the low-molecular-weight components produced via transesterification acted as plasticizers and softened the sample.

In contrast, the tan δ curve for PBS/GPR10 showed a distinct peak corresponding to the PCL side chains of GPR at approximately −60 °C, in addition to the T_g_ derived from PBS. The presence of two distinct T_g_ values indicated that PBS and GPR formed a macroscopically phase-separated structure.

For PBS/GPR10/Ti and PBS/GPR10/HDI, T_g_ derived from GPR shifted slightly toward that of PBS. This was possibly due to partial interfacial interaction between PBS and GPR via transesterification with Ti or urethanization with HDI that slightly enhanced the compatibility between different phases. For [PBS/GPR5/Ti]-HDI, [PBS/GPR10/HDI]-Ti, and [PBS/GPR10/Ti]-HDI, T_g_ derived from GPR approached that derived from PBS. These results confirmed that the two-step kneading method more effectively improved the compatibility between PBS and GPR due to efficiently progressing copolymerization.

### 3.5. Thermal Stability Evaluation via TGA

**Figure 9:**
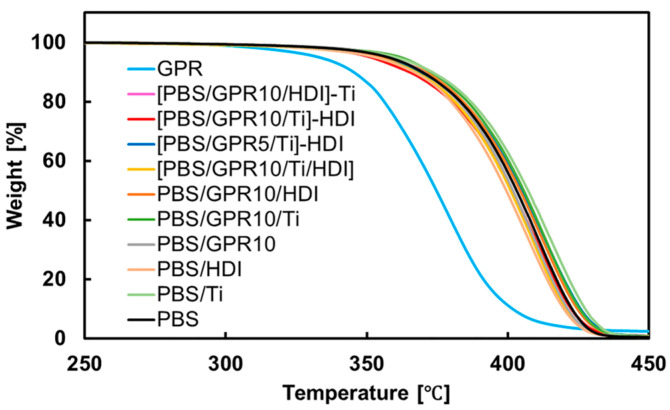
TGA curves of PBS, GPR, and representative blend samples measured under a nitrogen atmosphere.

Figure 9 shows the TG curves, and Table 7 presents the values of T_d,5%_ and T_max_ obtained from the TGA measurements. Although pure GPR exhibited lower T_d,5%_ and T_max_ values than pure PBS, no notable decrease in these thermal parameters was observed for any of the blend samples compared with unmodified PBS.

This behavior is attributed to the formation of PBS as a continuous matrix phase within the blend; therefore, the thermal decomposition behavior of the entire system is primarily dominated by the PBS matrix. Although the less thermally stable GPR is dispersed within the PBS, its content (10 wt%) is relatively low; consequently, it does not considerably compromise the overall weight loss behavior.

Furthermore, the DTG curves for all samples exhibited a single decomposition peak, with no distinct shoulders or multistep decomposition behavior observed. This indicates that the decomposition behavior of the major component, PBS, is dominant and that the decomposition temperature ranges of the individual components are close to each other. These results demonstrate that the PBS blend materials synthesized in this study maintain the excellent inherent thermal stability of PBS, even with the incorporation of the less heat-resistant GPR additive.

### 3.6. Morphology Characterizations via TEM

**Figure 10:**
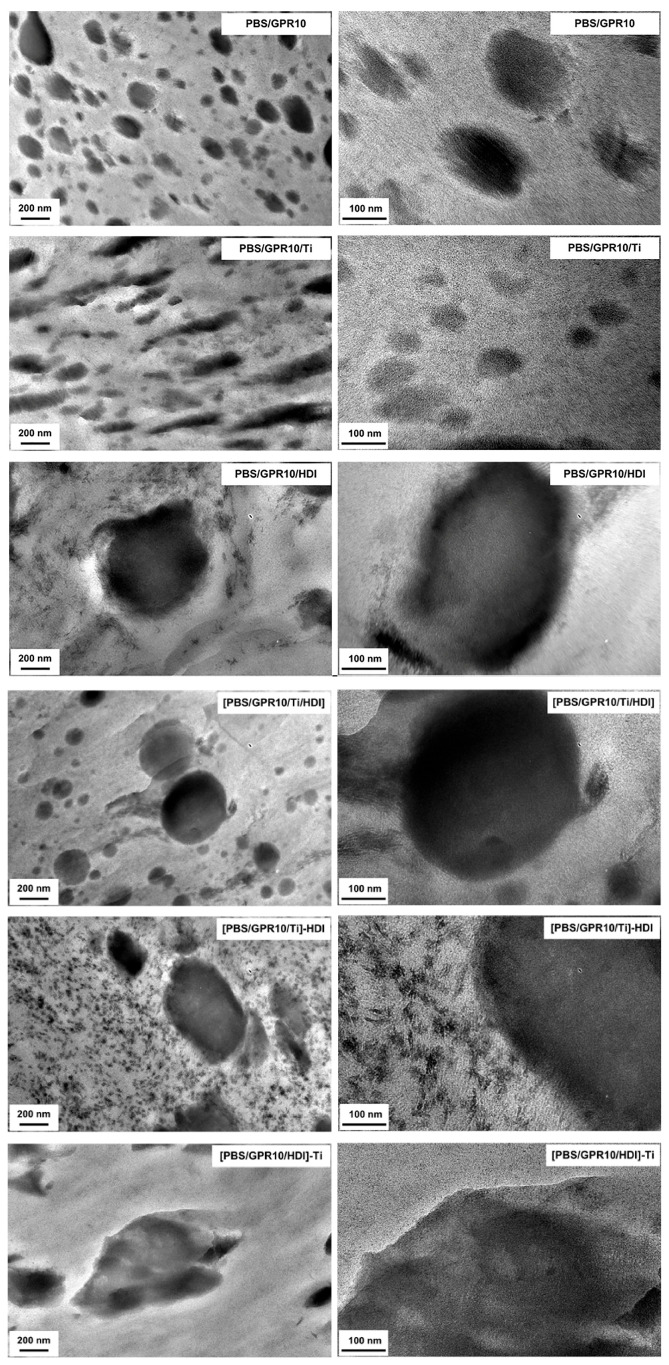
TEM images showing the morphology of PBS-based polymer blends.

Figure 10 shows the TEM images of the ultrathin sections of each sample, wherein the GPR phase appears dark due to RuO_4_ staining.

In PBS/GPR10, spherical GPR domains of ~100-nm diameter was uniformly dispersed in the PBS matrix. In contrast, PBS/GPR10/HDI and [PBS/GPR10/Ti/HDI] exhibited larger domains with a diameter of 200–300 nm because the addition of HDI promoted crosslinking between GPR molecules, suppressing domain breakup. In contrast, [PBS/GPR10/Ti]-HDI exhibited a characteristic structure comprising both fine domains (several tens of nm) and coarse domains (200–300 nm). These fine domains were formed because GPR molecules grafted onto PBS during the first-step transesterification acted as “anchors.” As a result, the second-step urethanization proceeded efficiently at the PBS interface and formed finely dispersed GPR domains. The relatively large domains were unreacted GPR molecules remaining after the first step, which underwent gelation induced by HDI and prevented domain breakup.

In [PBS/GPR10/HDI]-Ti, where the reaction order was reversed, such fine domains were not formed. This was because crosslinking (gelation) between GPR molecules proceeded preferentially with HDI in the first step. Thus, even when Ti was added in the second step, the course GPR gel could not be dispersed.

### 3.7. Evaluation of Mechanical Properties

#### 3.7.1. Izod Impact Test

**Figure 11:**
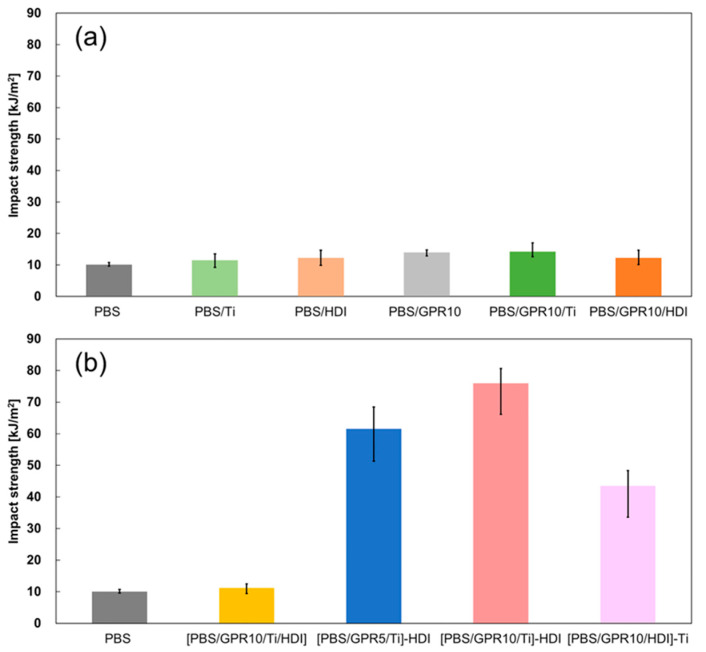
Izod impact strength of (**a**) untreated and one-step blended samples and (**b**) two-step blended samples.

Figure 11 shows the results of the notched Izod impact test, and Table 8 shows the corresponding key results as numerical values.

The impact strength of unmodified PBS was ~10 kJ/m^2^, and no significant improvement was observed in the impact strengths of PBS/Ti, PBS/GPR10, and PBS/GPR10/Ti. In contrast, the impact strength of [PBS/GPR10/Ti]-HDI reached 75.9 kJ/m^2^, approximately 7.5 times greater than that of unmodified PBS. The impact strengths of [PBS/GPR5/Ti]-HDI (61.6 kJ/m^2^) and [PBS/GPR10/HDI]-Ti (43.5 kJ/m^2^) also improved considerably due to a combination of factors. First, the GPR gel domains (observed in the TEM images) effectively inhibited crack propagation and absorbed impact energy. Second, the high interfacial adhesion (obtained via DMA measurements) considerably increased the energy consumption as cracks passed through the interface. Third, the formation of branched structures and increased molecular weight (indicated via rheological measurements) enabled load transfer and reduced stress concentration.

These findings indicated that the reaction sequence considerably influenced the impact strength and physical properties. However, even when HDI and Ti were added together, the impact strength of [PBS/GPR10/Ti/HDI] did not improve. Upon adding Ti, the impact strength of [PBS/GPR10/HDI]-Ti improved considerably; however, it did not match the impact strength of [PBS/GPR10/Ti]-HDI, in which Ti reacted first.

The relationship between dispersion state and impact strength is particularly noteworthy. Typically, improved dispersibility of the rubber component correlates with higher impact strength. However, PBS/GPR10 and PBS/GPR10/Ti, which showed relatively good dispersion, exhibited low impact strengths. This was due to poor crosslinking of GPR molecules, as confirmed via DSC, which deteriorated the functionality of GPR as a gel. Thus, fine dispersion of GPR molecules is not enough; the domain itself must function sufficiently as a “gel” that can absorb impact energy to toughen the material. Although GPR underwent crosslinking and its molecular weight increased upon adding HDI, DMA indicated that its T_g_ was low due to its enhanced impact energy–absorption capability.

#### 3.7.2. Morphological Observation of Impact Fracture Surfaces

**Figure 12:**
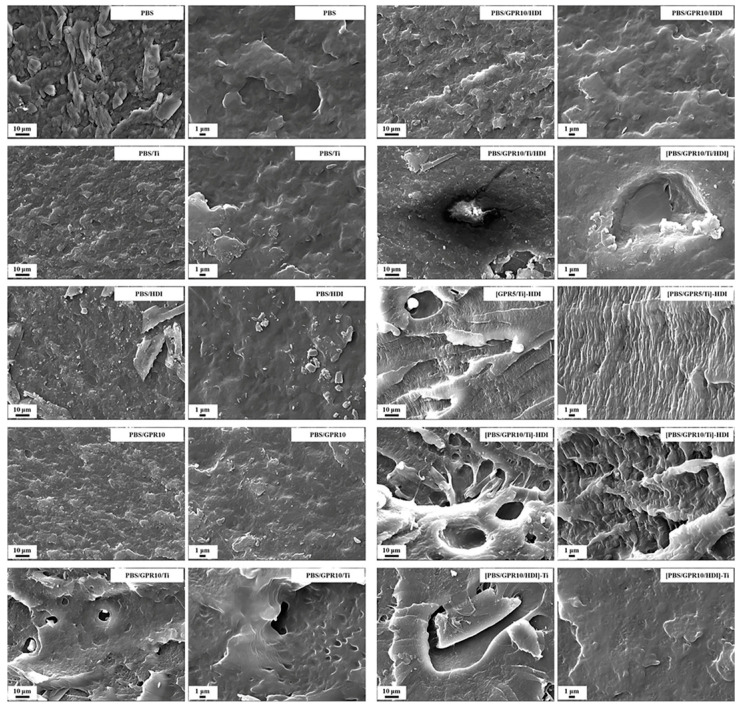
SEM images of fracture surfaces of samples after Izod impact testing.

Figure 12 shows the SEM images of fracture surfaces after the Izod impact test. Samples with low impact strength, such as unmodified PBS and PBS/GPR10/Ti, showed a relatively smooth fracture surface, which is typical of brittle fractures [62,63].

In contrast, the fracture surface of [PBS/GPR10/Ti]-HDI, which showed an extremely high impact strength, exhibited a different morphology: extensive stress whitening, with numerous fine crazes and traces of shear deformation. These features are characteristic of ductile fracture and indicate that the material underwent significant plastic deformation to absorb the impact energy. In addition, numerous fibril structures, in which the matrix resin stretched from the GPR domain interface, were observed. These observations confirmed the excellent interfacial adhesion between the two phases [64,65].

Samples with insufficient interfacial control showed characteristic fracture morphologies. In [PBS/GPR10/Ti/HDI] and PBS/GPR10/HDI, clear debonding was observed at the interface between the GPR domain and the matrix. This indicates low interfacial adhesion and that the domains acted as stress-concentration points during impact and became the origin of fracture.

#### 3.7.3. Tensile Test

**Figure 13:**
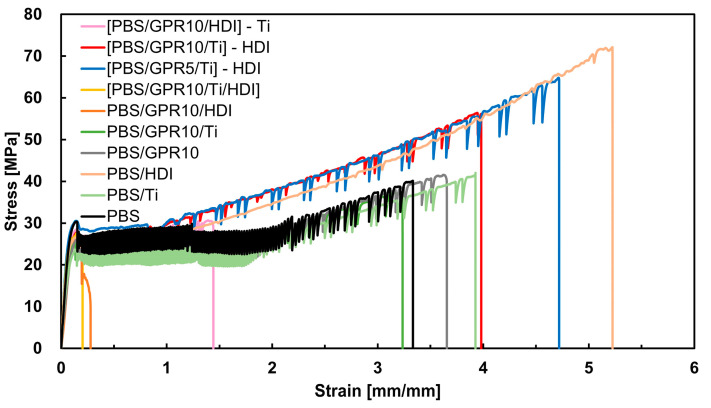
Representative stress–strain curves of samples derived via tensile tests.

Figure 13 shows the stress–strain curves derived via the tensile test. Table 9 summarizes the strain at break (ε_b_), stress at break (σ_b_), yield strength (σ_y_), tensile modulus (E), and energy to break (W_f_) for each sample.

In the PBS/Ti sample, the low-molecular-weight PBS generated as a by-product of transesterification functioned as a plasticizer; thus, this sample exhibited improved elongation at break and decreased tensile modulus compared with those of unmodified PBS. PBS/HDI exhibited considerably higher elongation at break compared with unmodified PBS due to decreased crystallinity (i.e., an increase in the amorphous region), as suggested via DSC measurements, and increased molecular weight due to the addition of HDI.

[PBS/GPR5/Ti]-HDI and [PBS/GPR10/Ti]-HDI exhibited considerably lower elongation at break compared with PBS/HDI. However, the former samples exhibited notable strain hardening behavior after yielding and maintained a high yield stress. As a result, their toughness (energy absorbed until break), defined by the area under the stress–strain curve, was more than twice that of unmodified PBS. This strain hardening was presumed to occur because the crosslinked domains formed by GPR alleviated stress concentration during stretching and suppressed the onset of localized necking. PBS/GPR10/HDI, [PBS/GPR10/Ti/HDI], and [PBS/GPR10/HDI]-Ti fractured at low strain. This brittle fracture was attributed to the low interfacial adhesion between the PBS matrix and GPR. In other words, Ti was not present in PBS/GPR10/HDI to initiate interfacial reactions and HDI in [PBS/GPR10/Ti/HDI] and [PBS/GPR10/HDI]-Ti hindered these reactions. Such a weak interface became the origin of fracture.

### 3.8. Gel Fraction Measurement

The underlying mechanism of improved mechanical properties of samples was elucidated by determining their gel fractions. The corresponding results are summarized in Table 10, along with the Izod impact strength, viscosity, tensile test results, and W_f_ and ε_b_.

Gelation did not occur in the samples containing only, even after adding Ti or HDI. However, the viscosity of the HDI-containing sample considerably increased due to chain extension of the PBS matrix. As the PBS-only sample did not contain GPR, this chain-extension effect of HDI was exhibited directly. However, gelation did not occur in GPR-containing samples upon adding Ti. A gel component was formed upon adding HDI (PBS/GPR10/HDI) due to the crosslinking of GPR. These findings indicated that gelation mainly occurred due to crosslinking of GPR by HDI. The viscosity of GPR-containing samples also increased upon adding HDI but to a smaller extent compared with that of PBS-only samples. This was because HDI added to the GPR-containing samples was consumed in competing reactions: “crosslinking of GPR” and “chain extension of PBS.” As part of HDI was used in the reaction with GPR, less HDI was available for PBS chain extension; this suppressed the increase in matrix viscosity.

Notably, a high gel fraction does not necessarily yield high impact strength. PBS/GPR10/HDI and [PBS/GPR10/Ti/HDI] exhibited high gel fractions; however, their impact strengths were similar to that of unmodified PBS. TEM and SEM observations revealed that the crosslinking of GPR by HDI occurred before the Ti catalyst could facilitate an interfacial reaction; this ultimately led to insufficient adhesion between PBS and GPR. In contrast, [PBS/GPR5/Ti]-HDI and [PBS/GPR10/Ti]-HDI exhibited extremely high impact strength despite having a gel fraction lower than the GPR content. This was due to the following ideal reaction sequence. In the first step, the interface of PBS and GPR chemically bonded due to Ti-catalyzed transesterification. HDI added in the second step promoted the toughening of the PBS matrix and the gelation of GPR domains while preserving the formed strong interface.

The lower impact strength of [PBS/GPR10/HDI]-Ti compared with [PBS/GPR10/Ti]-HDI, despite the reversed reaction order, also supported the aforementioned mechanism. As GPR underwent gelation and formed crosslinked domains in the first step, the interface could not be sufficiently modified upon the addition of Ti in the second step, resulting in moderate performance. Thus, the impact resistance of PBS can be considerably improved using GPR via an appropriate sequence of multiple reactions, rather than just a single reaction. The observed synergistic toughening resulted from a hierarchical reaction design: (1) the formation of a strong chemical bond at the PBS/GPR interface via a Ti-catalyzed transesterification and (2) simultaneous promotion of the internal crosslinking (gelation) of GPR domains and chain extension of the PBS matrix with HDI.

### 3.9. Biodegradability Evaluation

**Figure 14:**
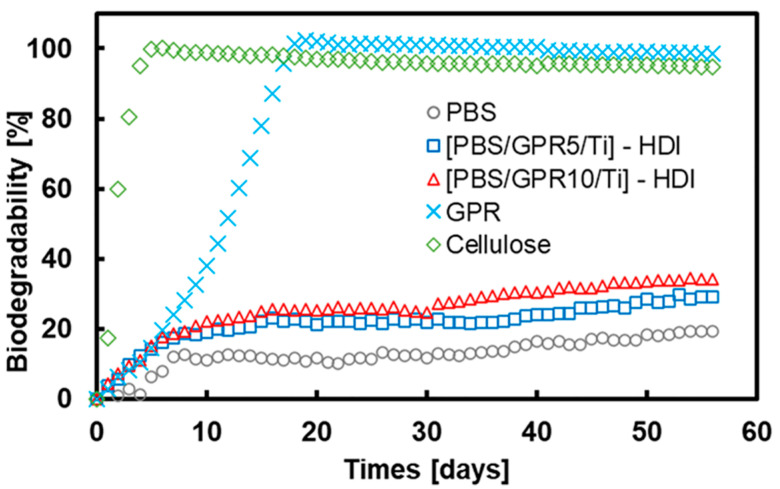
Biodegradability of samples evaluated using the BOD method over 56 days.

Figure 14 shows the results of the biodegradability test conducted using the BOD method. Cellulose, used as a positive control, began to decompose after ~20 days; after its decomposition, the oxygen consumption plateaued. GPR alone exhibited degradation behavior almost identical to that of cellulose, and its final oxygen consumption also plateaued. These findings confirmed the excellent biodegradability of GPR.

The biodegradability of PBS and GPR blends was clearly improved compared with that of PBS alone. Oxygen consumption also increased with increasing GPR content in these blends. This indicated that incorporating the easily degradable GPR into the PBS matrix enhanced the biodegradability of the material, mainly due to the selective degradation of GPR. This degradation facilitated the formation of a porous structure within the material, which possibly promoted its self-degradation by increasing the specific surface area of the PBS matrix.

**Figure 15:**
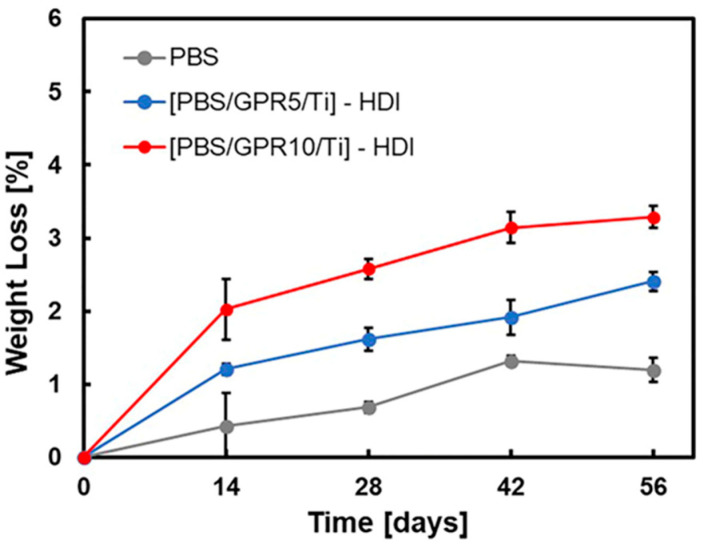
Weight loss (%) of samples in seawater degradation tests over 56 days, measured at 14-day intervals.

**Figure 16:**
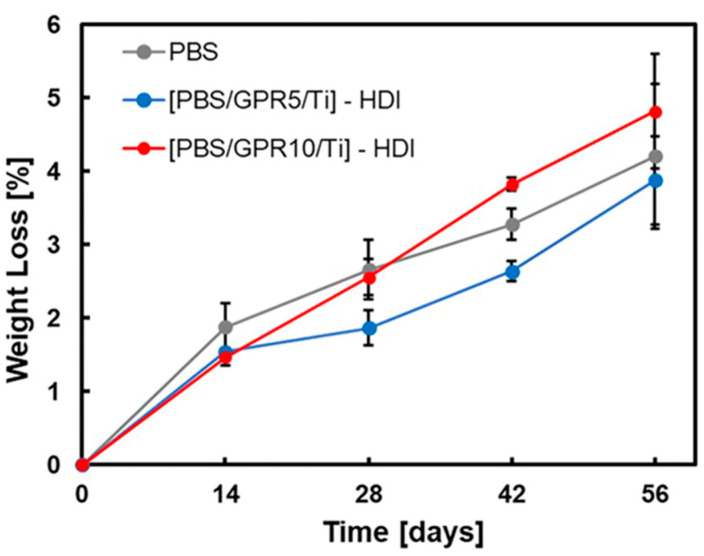
Weight loss (%) of samples in soil degradation tests over 56 days, measured at 14-day intervals.

Figure 15 and Figure 16 show the results of the disintegration tests performed in seawater and soil, respectively. In the marine environment test, consistent with the BOD test results, an increase in weight loss rate was observed with increasing GPR content. This indicated that introducing easily degradable GPR promoted the disintegration of the material in marine environments. All samples exhibited degradation behavior in soil, exhibiting no significant differences with the degradation behavior in seawater. This was likely because the PBS matrix exhibited excellent soil biodegradability; therefore, the presence or absence of GPR and differences in reaction conditions did not considerably impact its disintegration behavior.

In summary, reactive kneading can effectively enhance the degradability of samples in marine environments without impairing the inherent excellent soil biodegradability of PBS. Although the material exhibits enhanced biodegradability in microbe-rich marine environments, the PBS matrix is hydrophobic and is considered to remain structurally stable under typical ambient conditions. Therefore, the enhanced degradability is not expected to considerably compromise mechanical stability during the standard service life of the product.

## 4. Conclusions

In this study, the mechanical toughness of PBS was considerably improved using a two-step kneading method using GPR as the modifier. The impact strength of [PBS/GPR10/Ti]-HDI was considerably high, approximately 7.5 times that of unmodified PBS. [PBS/GPR10/Ti]-HDI was synthesized via Ti-catalyzed transesterification, followed by urethanization with HDI.

The mechanism underlying this high toughness was elucidated via multifaceted analyses, including FT-IR, DSC, DMA, TEM, and SEM. The improvement in toughness relied on three elements: (1) the formation of chemical bonds at the PBS/GPR interface, (2) the gelation of GPR domains, and (3) the toughening (chain extension) of the PBS matrix. Importantly, these processes must proceed in the correct sequence. The hierarchical reaction design, i.e., forming a strong interface via Ti-catalyzed transesterification, followed by the simultaneous strengthening of the matrix and domains by HDI, synergistically improved the mechanical properties of the sample. Reversing the reaction order or kneading in one step did not yield this pronounced effect, suggesting that precise control of the reaction process is essential.

This modification strategy also improved the degradability of samples in the marine environment without impairing the inherent excellent soil biodegradability of PBS. These findings indicate that the hierarchical reaction design is an effective strategy for achieving both biodegradability and high strength/toughness. This study opens new pathways for expanding the use of biodegradable plastics in fields requiring high durability, wherein their application has been limited. It also serves as an important guideline for realizing a sustainable society.

## Figures and Tables

**Table 1:** Composition ratios of the first-step blends of PBS, GPR, and additives.

Code	PBS [wt%]	GPR [wt%]	Ti [phr]	HDI [phr]
PBS	100	-	-	-
PBS/Ti	100	-	0.1	-
PBS/HDI	100	-	-	1
PBS/GPR10	90	10	-	-
PBS/GPR5/Ti	95	5	0.1	-
PBS/GPR10/Ti	90	10	0.1	-
PBS/GPR10/HDI	90	10	-	1
[PBS/GPR10/Ti/HDI]	90	10	0.1	1

**Table 2:** Composition ratios of the second-step blends with sequentially added additives based on first-step blends.

Code	Ti [phr]	HDI [phr]	PBS/GPR5/Ti [wt%]	PBS/GPR10/Ti [wt%]	PBS/GPR10/HDI [wt%]
[PBS/GPR5/Ti]-HDI	-	1	100	-	-
[PBS/GPR10/Ti]-HDI	-	1	-	100	-
[PBS/GPR10/HDI]-Ti	0.1	-	-	-	100

**Table 3:** Set temperatures in Sections 1–6, H/AD, and D.

Section	Section 1	Section 2	Section 3	Section 4	Section 5	Section 6	H/AD	D
Temperature [°C]	40	135	135	135	135	135	135	135

**Table 4:** Temperature in Section 4 and pressure measured at H/AD.

Code	Temperature [°C] at Section 4	Pressure [MPa] at H/AD
PBS	142	5.0
PBS/Ti	142	5.0
PBS/HDI	143	7.2
PBS/GPR10	136	4.9
PBS/GPR10/Ti	138	4.9
PBS/GPR10/HDI	138	5.9
[PBS/GPR10/Ti/HDI]	135	5.5
[PBS/GPR5/Ti]-HDI	135	6.2
[PBS/GPR10/Ti]-HDI	135	5.2
[PBS/GPR10/HDI]-Ti	135	5.9

**Table 5:** Thermal properties of PBS and GPR: T_m_, X_c_, and T_g_ for PBS; T_m_ and ΔH_m_ for GPR.

Code	PBS			GPR	
	T_m_ [°C]	χ_c_ [%]	T_g_ [°C]	T_m_ [°C]	ΔH_m_ [J/g]
PBS	112.9	59.3	−31.7	-	-
PBS/Ti	111.1	59.0	−33.4	-	-
PBS/HDI	109.5	54.8	−33.4	-	-
PBS/GPR10	112.2	58.8	−31.1	37.9	3.6
PBS/GPR10/Ti	111.6	59.8	−32.4	37.7	3.2
PBS/GPR10/HDI	111.0	54.5	−32.2	-	-
[PBS/GPR10/Ti/HDI]	110.8	60.8	−34.2	-	-
[PBS/GPR10/Ti]-HDI	111.6	55.9	−34.2	-	-
[PBS/GPR10/HDI]-Ti	110.6	57.4	−33.5	-	-

**Table 6:** T_g_ of PBS and GPR samples determined via DMA.

Code	PBS T_g_ [°C]	GPR T_g_ [°C]
PBS	−25.7	-
PBS/Ti	−28.6	-
PBS/HDI	−26.2	-
PBS/GPR10	−24.9	−56.1
PBS/GPR10/Ti	−25.4	−51.0
PBS/GPR10/HDI	−25.2	−52.5
[PBS/GPR10/Ti/HDI]	−24.3	−53.7
[PBS/GPR10/Ti]-HDI	−29.7	−49.4
[PBS/GPR10/HDI]-Ti	−27.1	−51.6

**Table 7:** Thermal decomposition properties of PBS, GPR, and blend samples: 5% weight loss temperature (T_d,5%_) and maximum decomposition temperature (T_max_).

Code	T_d,5%_ [°C]	T_max_ [°C]
PBS	355.1	413.5
GPR	336.7	380.1
PBS/Ti	357.9	414.8
PBS/HDI	356.3	412.8
PBS/GPR10	357.2	416.1
PBS/GPR10/Ti	353.0	416.8
PBS/GPR10/HDI	355.1	413.1
[PBS/GPR10/Ti/HDI]	354.5	413.2
[PBS/GPR5/Ti]–HDI	357.1	414.5
[PBS/GPR10/Ti]–HDI	356.3	413.4
[PBS/GPR10/HDI]–Ti	355.1	413.1

**Table 8:** Izod impact strength of PBS-based polymer blends.

Code	Impact Strength [kJ/m^2^]
PBS	10.1
PBS/Ti	11.5
PBS/HDI	12.3
PBS/GPR10	14.0
PBS/GPR10/Ti	14.3
PBS/GPR10/HDI	12.3
[PBS/GPR10/Ti/HDI]	11.2
[PBS/GPR5/Ti]-HDI	61.6
[PBS/GPR10/Ti]-HDI	75.9
[PBS/GPR10/HDI]-Ti	43.5

**Table 9:** Mechanical properties of samples determined via tensile tests: ε_b_, σ_y_, σ_b_, E, and W_f_.

Code	ε_b_ [mm/mm]	σ_b_ [MPa]	σ_y_ [MPa]	E [MPa]	W_f_ [MJ/m^3^]
PBS	3.35	38.4	28.0	423	89.8
PBS/Ti	3.85	40.7	23.6	267	106
PBS/HDI	5.10	68.8	26.0	321	193
PBS/GPR10	3.68	40.9	25.3	351	103
PBS/GPR10/Ti	3.07	35.0	23.3	287	78.3
PBS/GPR10/HDI	0.27	23.6	23.2	261	4.85
[PBS/GPR10/Ti/HDI]	0.20	27.6	26.8	364	4.23
[PBS/GPR5/Ti]-HDI	4.53	64.0	29.4	480	188
[PBS/GPR10/Ti]-HDI	3.92	56.2	26.8	437	149
[PBS/GPR10/HDI]-Ti	1.51	32.2	28.5	295	41.0

**Table 10:** Mechanical properties of all samples: gel fraction, Izod impact strength, η* at 1 rad/s, W_f_, and ε_b_.

Code	Gel Fraction [%]	Impact Strength [kJ/m^2^]	η [Pa·s] @1 rad/s	W_f_ [kJ/m^2^]	ε_b_ [mm/mm]
PBS	0	10.1	7350.7	89.8	3.35
PBS/Ti	0	11.5	7280.6	106	3.85
PBS/HDI	0	12.3	64,433	193	5.10
PBS/GPR10	0	14.0	7372.1	103	3.68
PBS/GPR10/Ti	0	14.3	7590.5	78.3	3.07
PBS/GPR10/HDI	7.77	12.3	33,917	4.85	0.27
[PBS/GPR10/Ti/HDI]	9.83	11.2	15,895	4.23	0.20
[PBS/GPR5/Ti]-HDI	0.99	61.6	38,481	188	4.53
[PBS/GPR10/Ti]-HDI	3.07	75.9	30,619	149	3.92
[PBS/GPR10/HDI]-Ti	7.31	43.5	32,647	41.0	1.51

## Data Availability

The original contributions presented in this study are included in the article. Further inquiries can be directed to the corresponding author.

## Abbreviations

The following abbreviations are used in this manuscript:

PBS	Poly(butylene succinate)
GPR	Grafted polyrotaxane
PR	Polyrotaxane
PCL	Polycaprolactone
HDI	Hexamethylene diisocyanate
E′	Storage modulus
DSC	Differential scanning calorimetry
FT-IR	Fourier transform-infrared spectroscopy
DMA	Dynamic mechanical analysis
TEM	Transmission electron microscopy
SEM	Scanning electron microscopy
BOD	Biochemical oxygen demand
ThOD	Theoretical oxygen demand
T_g_	Glass-transition temperature
T_m_	Melting temperature
W_f_	Energy to break
SDS	Sodium dodecyl sulfate
η*	Complex viscosity
